# Follow-up Survey of US Adult Reports of Mental Health, Substance Use, and Suicidal Ideation During the COVID-19 Pandemic, September 2020

**DOI:** 10.1001/jamanetworkopen.2020.37665

**Published:** 2021-02-19

**Authors:** Mark É. Czeisler, Rashon I. Lane, Joshua F. Wiley, Charles A. Czeisler, Mark E. Howard, Shantha M. W. Rajaratnam

**Affiliations:** 1 Turner Institute for Brain and Mental Health, Monash University, Clayton, Victoria, Australia; 2 US Centers for Disease Control and Prevention, Atlanta, Georgia; 3 Division of Sleep and Circadian Disorders, Departments of Medicine and Neurology, Brigham and Women’s Hospital, Harvard Medical School, Boston, Massachusetts; 4 Institute for Breathing and Sleep, Austin Health, Heidelberg, Victoria, Australia

## Abstract

This survey study compared patterns of mental health concerns, substance use, and suicidal ideation during June and September 2020 of the COVID-19 pandemic and examined at-risk demographic groups.

## Introduction

Adverse mental health symptoms among US adults were more prevalent during the early phase (April-June 2020) of the coronavirus disease 2019 (COVID-19) pandemic compared with prepandemic estimates (eg, 3-fold increased prevalences of anxiety and depression symptoms, 2-fold increased prevalence of suicidal ideation).^1,2^ In June 2020, 2238 (40.9%) of 5470 US adults reported adverse mental or behavioral health symptoms. During this time, the prevalence of symptoms was lower in adults aged 65 years or older (141 of 933 [15.1%]) than in young adults aged 18 to 24 years (547 of 731 [74.9%]; *P* < .001).^1^ Given suggestions that acute increases in the prevalence of adverse mental health symptoms may represent a transient response to mass trauma,^3^ we sought to determine whether these patterns persisted in September 2020 and to examine disproportionately affected demographic groups.

## Methods

In this survey study from August 28 to September 6, 2020, US adults aged 18 years or older completed 139-item internet-based surveys through Qualtrics for The COVID-19 Outbreak Public Evaluation (COPE) Initiative. Surveys were administered to an online respondent panel maintained by Qualtrics, a commercial survey company with networks of participant pools. Respondents reported demographic characteristics and completed questions assessing attitudes, behaviors, and beliefs about COVID-19, mitigation measures, and mental and behavioral health. When possible, brief, validated instruments were used or adapted.

Demographic quota sampling and survey weighting were used to make the sample representative of the US population by age, sex, and race/ethnicity, and weighted values are presented. Participants reported symptoms of anxiety and depression, COVID-19−related trauma- and stressor-related disorders, starting or increasing substance use to cope with pandemic-related stress, or having seriously considered suicide within 30 days. The Monash University Human Research Ethics Committee approved the study protocol, and participants provided informed consent electronically. The article followed the American Association for Public Opinion Research (AAPOR) reporting guideline.

Multivariable Poisson regressions with robust standard errors were used to estimate adjusted prevalence ratios (aPRs) and 95% CIs for any adverse mental or behavioral health symptom with the following factors: sex, age, sexual orientation, race/ethnicity, Census region, urban/rural residence, and unpaid caregiver status. Separate models were run for the following collinear factors: disability status, insomnia symptoms, prior psychiatric diagnosis (anxiety, depression, posttraumatic stress disorder, or a substance use disorder), and age-excluded employment status. Age was not adjusted for in the model that included employment status to avoid collinearity between these variables. Continuity-corrected McNemar tests were used to assess longitudinal differences in adverse mental health symptom prevalences among respondents who completed surveys in June 2020 and September 2020. All calculations were performed in Python version 3.7.8 (Python Software Foundation) and R version 4.0.2 (The R Project for Statistical Computing) using the R survey package version 3.29. *P* values were 2-sided, and statistical significance was set at *P* < .05. Detailed methods^1^ describing the recruitment process, survey, screening tools, and analyses can be found in the eAppendix in the Supplement.

## Results

Overall, 5285 of 11 953 potential participants (44.2%) completed September 2020 surveys; 5186 of these respondents (98.1%) met secondary screening criteria and were analyzed (1155 [22.3%] were recontacted after April 2020; 1605 [30.9%] were recontacted after June 2020; 2426 [46.8%] were first-time respondents). Overall, 1710 (33.0%) reported anxiety or depression symptoms, 1536 (29.6%) reported COVID-19–related trauma- and stressor-related disorder symptoms, 781 (15.1%) reported increased substance use, 618 (11.9%) reported having seriously considered trying to kill themselves in August, and 2237 (43.1%) reported at least 1 of these symptoms (Table 1).

**Table 1.** Prevalence of Adverse Mental and Behavioral Health Symptoms, by Respondent Characteristics

Characteristic	No. (%)					
	Total respondents	Anxiety or depression	COVID-19 TSRD	Substance use	Suicidal ideation	≥1 of these
June 2020^a^	5470 (100)	1692 (30.9)	1437 (26.3)	726 (13.3)	584 (10.7)	2238 (40.9)
September 2020	5186 (100)	1710 (33.0)	1536 (29.6)	781 (15.1)	618 (11.9)	2237 (43.1)
Sex						
Female	2641 (50.9)	887 (33.6)	764 (28.9)	327 (12.4)	240 (9.1)	1156 (43.8)
Male	2545 (49.1)	823 (32.3)	773 (30.4)	454 (17.8)	378 (14.9)	1081 (42.5)
Sexual orientation						
Heterosexual	4568 (88.1)	1373 (30.1)	1261 (27.6)	570 (12.5)	436 (9.5)	1818 (39.8)
Lesbian or gay	242 (4.7)	121 (50.0)	101 (41.9)	73 (30.4)	55 (22.7)	148 (61.1)
Bisexual	202 (3.9)	131 (64.8)	99 (48.8)	95 (47.1)	79 (39.0)	159 (78.6)
Other or unknown^b^	174 (3.4)	84 (48.5)	75 (43.3)	42 (24.1)	49 (28.0)	112 (64.2)
Age group, y						
18-24	593 (11.4)	376 (63.4)	309 (52.2)	168 (28.4)	118 (19.9)	441 (74.4)
25-44	1837 (35.4)	886 (48.2)	813 (44.2)	493 (26.8)	426 (23.2)	1122 (61.1)
45-64	1831 (35.3)	366 (20.0)	327 (17.8)	95 (5.2)	64 (3.5)	536 (29.3)
≥65	926 (17.9)	82 (8.9)	88 (9.5)	24 (2.6)	11 (1.2)	138 (14.9)
Race/ethnicity						
White non-Hispanic	3349 (64.6)	952 (28.4)	857 (25.6)	418 (12.5)	341 (10.2)	1238 (37.0)
Black non-Hispanic	634 (12.2)	244 (38.4)	243 (38.3)	117 (18.5)	92 (14.5)	346 (54.5)
Asian non-Hispanic	261 (5.0)	58 (22.3)	64 (24.6)	14 (5.3)	13 (4.8)	93 (35.7)
Other race or multiple races, non-Hispanic^c^	159 (3.1)	59 (36.8)	45 (28.1)	14 (8.7)	10 (6.6)	74 (46.7)
Hispanic, any race or races	782 (15.1)	397 (50.8)	328 (41.9)	218 (27.9)	163 (20.8)	486 (62.1)
Employment status						
Nonessential worker	1303 (25.1)	333 (25.5)	322 (24.7)	133 (10.2)	79 (6.1)	487 (37.4)
Essential worker	1767 (34.1)	876 (49.5)	805 (45.5)	536 (30.3)	472 (26.7)	1087 (61.5)
Unemployed	720 (13.9)	263 (36.5)	204 (28.3)	56 (7.8)	36 (4.9)	336 (46.8)
Retired	1242 (23.9)	161 (13.0)	142 (11.4)	42 (3.4)	17 (1.4)	239 (19.3)
Student	154 (3.0)	77 (49.8)	64 (41.2)	13 (8.1)	14 (9.2)	87 (56.2)
Unpaid caregiver status						
No	3259 (62.8)	705 (21.6)	632 (19.4)	163 (5.0)	93 (2.8)	1003 (30.8)
Children or adolescents <18 y	484 (9.3)	138 (28.6)	127 (26.1)	39 (8.0)	14 (3.0)	196 (40.4)
Adults ≥18 y	544 (10.5)	200 (36.7)	171 (31.5)	47 (8.6)	35 (6.4)	258 (47.4)
Both age groups	899 (17.3)	666 (74.1)	607 (67.5)	532 (59.2)	476 (53.0)	781 (86.8)
Disability status^d^						
Yes	1158 (22.3)	647 (55.9)	551 (47.6)	395 (34.1)	349 (30.1)	770 (66.5)
No	3812 (73.5)	982 (25.8)	926 (24.3)	365 (9.6)	262 (6.9)	1368 (35.9)
Prefer not to say	216 (4.2)	81 (37.3)	60 (27.5)	21 (9.6)	7 (3.2)	99 (45.7)
Symptoms of insomnia^e^						
Yes	899 (17.3)	512 (56.9)	460 (51.1)	202 (22.5)	170 (18.9)	617 (68.6)
No	4287 (82.7)	1197 (27.9)	1076 (25.1)	579 (13.5)	448 (10.5)	1621 (37.8)
Past psychiatric diagnosis^f^						
Yes	1919 (37.0)	1189 (62.0)	977 (50.9)	599 (31.2)	508 (26.5)	1380 (71.9)
No	3267 (63.0)	521 (15.9)	560 (17.1)	182 (5.6)	110 (3.4)	857 (26.2)

Abbreviations: COVID-19, coronavirus disease 2019; TSRD, trauma- and stressor-related disorder.

^a^ Data appeared in Czeisler et al,^1^ 2020.

^b^ Includes responses of something else, I don’t know the answer, and prefer not to say.

^c^ Includes American Indian or Alaskan Native, Native Hawaiian or other Pacific Islander, and other.

^d^ Includes physical, mental, and emotional conditions or health conditions that require special equipment.

^e^ Assessed via the 2-item Sleep Condition Indicator.

^f^ Includes current or prior diagnosis with an anxiety disorder, depression, posttraumatic stress disorder, or a substance use disorder.

Adverse mental or behavioral health symptoms were more prevalent among adults younger than 65 years vs adults aged 65 years or older (eg, 18-24 years, aPR, 3.56 [95% CI, 3.04-4.18]) and among multigenerational caregivers vs noncaregivers (aPR, 1.93 [95% CI, 1.78-2.08]) and respondents with prior psychiatric diagnoses vs those with no prior diagnoses (aPR, 1.98 [95% CI, 1.83-2.15]) (Table 2). Prevalence of adverse mental or behavioral health symptoms was also higher among respondents with disabilities or insomnia symptoms vs those without, caregivers for adults vs noncaregivers, essential workers and unemployed respondents vs nonessential workers, and respondents who were lesbian, gay, or bisexual vs heterosexual. Among respondents who were recontacted after June 2020, prevalence of adverse mental health symptoms did not differ significantly between June 2020 and September 2020.

**Table 2.** Characteristics Associated With Adverse Mental or Behavioral Health Symptoms, September 2020

Characteristic	aPR (95% CI)^a^
Age group, y	
≥65	1 [Reference]
18-24	3.56 (3.04-4.18)
25-44	3.15 (2.76-3.60)
45-64	1.81 (1.58-2.07)
Sexual orientation	
Heterosexual	1 [Reference]
Lesbian or gay	1.25 (1.13-1.39)
Bisexual	1.26 (1.14-1.39)
Employment^b^	
Nonessential worker	1 [Reference]
Essential worker	1.28 (1.17-1.41)
Unemployed	1.24 (1.10-1.40)
Student	1.28 (1.00-1.66)
Retired	0.59 (0.52-0.67)
Unpaid caregiver status	
No	1 [Reference]
Children or adolescents <18 y	1.06 (0.92-1.21)
Adults ≥18 y	1.38 (1.24-1.54)
Both age groups	1.93 (1.78-2.08)
Disability status^c^	
No	1 [Reference]
Yes	1.40 (1.30-1.49)
Symptoms of insomnia^d^	
No	1 [Reference]
Yes	1.64 (1.54-1.75)
Past psychiatric diagnosis^e^	
No	1 [Reference]
Yes	1.98 (1.83-2.15)

Abbreviation: aPR, adjusted prevalence ratio.

^a^ Adjusted for sex, age, sexual orientation, race/ethnicity, region, urban/rural residence, and unpaid caregiver status. Groups without significant aPR estimates are not shown.

^b^ Age was not adjusted for in the employment status model to avoid collinearity between these variables.

^c^ Includes physical, mental, and emotional conditions or health conditions that require special equipment.

^d^ Assessed via the 2-item Sleep Condition Indicator.

^e^ Includes current or prior diagnosis with an anxiety disorder, depression, posttraumatic stress disorder, or a substance use disorder.

## Discussion

In a later phase of the COVID-19 pandemic (September 2020), the prevalence of adverse mental health symptoms among US adults remained elevated compared with prepandemic estimates.^1,2^ This finding contradicts the notion that adverse mental health symptoms were transient, self-limiting responses. Despite increased COVID-19–related morbidity and mortality risk,^4^ adverse mental health symptoms among older adults remained less prevalent.^1,2,5,6^ Although quota sampling and survey weighting were used, internet-based survey samples are limited and may not fully represent the 2020 US population.^1^ Nonetheless, evidence of sustained adverse mental health symptoms among more than 5000 community-dwelling US adults highlights the need to promote preventive behaviors, expand mental health care access, and integrate medical and behavioral health services to mitigate the mental health effects of COVID-19.

## Notes

Supplement. **eAppendix.** Supplementary Methods **eReferences.**
