# Supplementary material for: Follow-up Survey of US Adult Reports of Mental Health, Substance Use, and Suicidal Ideation During the COVID-19 Pandemic, September 2020
Source: JAMA Netw Open. 2021 Feb 19;4(2):e2037665. doi: 10.1001/jamanetworkopen.2020.37665 (PMC7896196; doi:10.1001/jamanetworkopen.2020.37665)
Supplement: Supplement. — eAppendix. Supplementary Methods eReferences. [file jamanetwopen-e2037665-s001.pdf]

Supplemental Online Content

Czeisler MÉ, Lane RI, Wiley JF, Czeisler CA, Howard ME, Rajaratnam SMW. Follow-up survey of US adult reports of mental health, substance use, and suicidal ideation during the COVID-19 pandemic, September 2020. *JAMA Netw Open*. 2021;4(2):e2037665. doi:10.1001/jamanetworkopen.2020.37665

eAppendix. Supplementary Methods

eReferences.

This supplemental material has been provided by the authors to give readers additional information about their work.

© 2021 Czeisler MÉ et al. *JAMA Network Open.*

eAppendix. Supplementary Methods

Study review and approval

This activity was reviewed by US Centers for Disease Control and Prevention (CDC) and was conducted consistent with applicable federal law and CDC policy (i.e., 45 C.F.R. part 46, 21 C.F.R. part 56; 42 U.S.C. Sect. 241(d); 5 U.S.C. Sect. 552a; 44 U.S.C. Sect. 3501 et seq.).

Recruitment Methodologies

Qualtrics recruitment methodologies include digital advertisements and promotions, word of mouth, and membership referrals, social networks, television and radio advertisements, and offline, mail-based approaches. Potential respondents received invitations and could opt to participate by activating a survey link directing them to the participation information and consent page preceding the survey. Ineligible respondents who did not meet inclusion criteria (eg, aged below 18 years, or exceeded pre-specified demographic quotas) were disempaneled from the survey.

Screening tools

Symptoms of anxiety and depression were assessed via the 4-item Patient Health Questionnaire (PHQ-4).[1] Those who scored at least 3 of 6 on the Generalized Anxiety Disorder (GAD-2) or the Patient Health Questionnaire (PHQ-2) subscales were considered symptomatic of an anxiety or depression. Symptoms of a COVID-19 trauma- and stressor-related disorder (TSRD) were assessed via the 6-item Impact-of-Event Scale to screen for overlapping symptoms of posttraumatic stress disorder, acute stress disorder, and adjustment disorders (IES-6).[2] For this survey, the COVID-19 pandemic

© 2021 Czeisler MÉ et al. *JAMA Network Open.*

was specified as the traumatic exposure to record peri- and posttraumatic symptoms associated with the range of stressors introduced by the COVID-19 pandemic. Those who scored at least 1.75 of 4 were considered symptomatic. Trauma- and stressor-related symptoms assessed were common to multiple TSRDs, including posttraumatic stress disorder (PTSD), acute stress disorder (ASD), and adjustment disorders (ADs) to capture COVID-19–specific trauma and stress symptoms responsive to prolonged exposures that do not meet diagnostic criteria for PTSD.[3,4] Symptoms of insomnia were assessed via the 2-item Sleep Condition Indicator (SCI-02).[5] Those who scored less than or equal to 2 of 8 were considered symptomatic. Persons who had disabilities were defined as such based on a response indicating limitations of activities because of physical, mental, or emotional conditions, or health conditions that require special equipment, based on the CDC Behavioral Risk Factor Surveillance System. Substance use was defined as use of “alcohol, legal or illegal drugs, or prescriptions drugs that are taken in a way not recommended by your doctor,” and respondents were given the opportunity to respond “Yes”, “No”, or “Prefer not to say.” Suicidal ideation was indicated by responses to the question: “At any time in the past 30 days, did you seriously think about trying to kill yourself?”

Reporting Race/Ethnicity

Race and ethnicity were assessed among survey respondents with separate questions and options defined by the investigators based on US Census classifications. The race and ethnicity questions follow.

1. What is your race? (Select all that apply)
    a. American Indian or Alaskan Native

© 2021 Czeisler MÉ et al. *JAMA Network Open.*

b. Asian
c. Black or African American
d. Native Hawaiian or other Pacific Islander
e. White
f. Other

Please use the categories that most reflect your recognition in the community for purposes of reporting mixed racial and/or ethnic origins.
American Indian or Alaskan Native: a person having origins in any of the original peoples of North, Central, or South America, and maintains tribal affiliations or community attachment.
Asian: A person having origins in any of the original peoples of the Far East, Southeast Asia, or the Indian subcontinent including, for example, Cambodia, China, India, Japan, Korea, Malaysia, Pakistan, the Philippine Islands, Thailand, and Vietnam.
Black or African American: A person having origins in any of the black racial groups of Africa.
Native Hawaiian or Pacific Islander: A person having origins in any of the original peoples of Hawaii, Guam, Samoa, or other Pacific Islands.
White: A person having origins in any of the original peoples of Europe, North Africa, or the Middle East.

2. What is your ethnicity? (Select one)
   a. Hispanic or Latino
   b. Not Hispanic or Latino

Hispanic or Latino: A person of Cuban, Mexican, Puerto Rican, South or Central American, or other Spanish culture or origin regardless of race.

For this analysis, race and ethnicity were combined into the following categories: White, non-Hispanic; Black, non-Hispanic; Asian, non-Hispanic; Multiple races or Other race, non-Hispanic; and Hispanic, any race or races.

Race and ethnicity were assessed in this study given mental health disparities documented during the COVID-19 pandemic.

Secondary screening criteria

Following Qualtrics standard screening procedures, the investigators conducted a secondary screening, including removal of duplicate respondents, those who reported

© 2021 Czeisler MÉ et al. *JAMA Network Open.*

invalid US ZIP codes according to the US Department of Housing and Urban Development United States Postal Service ZIP Code Crosswalk Files, recontacts who had moved outside of the US, and those with missing or uncharacterizable responses to demographic questions used for weighting (sex, age, race/ethnicity).

Survey Weighting

Survey weights were trimmed ($0.3 \leq weight \leq 3.0$).

Longitudinal analysis, June 2020 and September 2020

Participants who completed June 2020 surveys, including first-time June 2020 respondents and those recontacted from April 2020, were reweighted for longitudinal analyses. McNemar χ^2 test with continuity correction was used to test for changes in prevalence of all 5 mental or behavioral health measures between June 2020 and September 2020.

September 2020 regressions

For regression models, respondents who did not provide characterizable responses to variables included in the model were excluded, including the following: sexual orientation (“Something else,” “I don’t know the answer,” and “Prefer not to say”; total n = 174 [3.4%]) and disability status (“Prefer not to say”; n = 216 [4.2%]).

Additional information

© 2021 Czeisler MÉ et al. *JAMA Network Open.*

Methods were further detailed elsewhere.[6]

© 2021 Czeisler MÉ et al. *JAMA Network Open.*

eReferences

1. Löwe B, Wahl I, Rose M, et al. A 4-item measure of depression and anxiety: validation and standardization of the Patient Health Questionnaire-4 (PHQ-4) in the general population. *J Affect Disord.* 2010;122:86-95. doi:10.1016/j.jad.2009.06.019

2. Hosey MM, Leoutsakos JS, Li X, et al. Screening for posttraumatic stress disorder in ARDS survivors: validation of the Impact of Event Scale-6 (IES-6). *Crit Care*. 2019;23:276. doi:10.1186/s13054-019-2553-z

3. Shevlin M, Hyland P, Karatzias T. Is posttraumatic stress disorder meaningful in the context of the COVID-19 pandemic: response to Van Overmeire's commentary on Karatzias et al. (2020). *J Trauma Stress*. 2020;3(5):866-868. doi:10.1002/jts.22592

4. Horesh D, Brown AD. Traumatic stress in the age of COVID-19: call to close critical gaps and adapt to new realities. *Psychol Trauma*. 2020;12:331-5. doi:2020-25108-001

5. Espie CA, Kyle SD, Hames P, et al. The Sleep Condition Indicator: a clinical screening tool to evaluate insomnia disorder. *BMJ Open*. 2014;4:e004183. doi:10.1136/bmjopen-2013-004183

6. Czeisler MÉ, Lane RI, Petrosky E, et al. Mental health, substance use, and suicidal ideation during the COVID-19 pandemic. *MMWR Morb Mortal Wkly Rep*. 2020;69(32):1049-1057. doi:10.15585/mmwr.mm6932a1

© 2021 Czeisler MÉ et al. *JAMA Network Open.*
